# Supplementary material for: Predicting visual function by interpreting a neuronal wiring diagram
Source: Nature. 2024 Oct 2;634(8032):113–23. doi: 10.1038/s41586-024-07953-5 (PMC11446822; doi:10.1038/s41586-024-07953-5)
Supplement: Supplementary file 6 — CRF and ERF predictions for individual TmY4 and TmY9 cells. Analogous to Supplementary Data 3, but for TmY target types. Shown are the top four monosynaptic pathways, the strongest pathway passing through each of the top ten intermediary types (ranking from Extended Data Fig. 7), and the trisynaptic pathway Tm1–TmY–Dm3–TmY (see the section entitled Prediction of spatial normalization). [file 41586_2024_7953_MOESM6_ESM.zip › DataS4/TmY9qΓèÑ/720575940637351525.pdf]

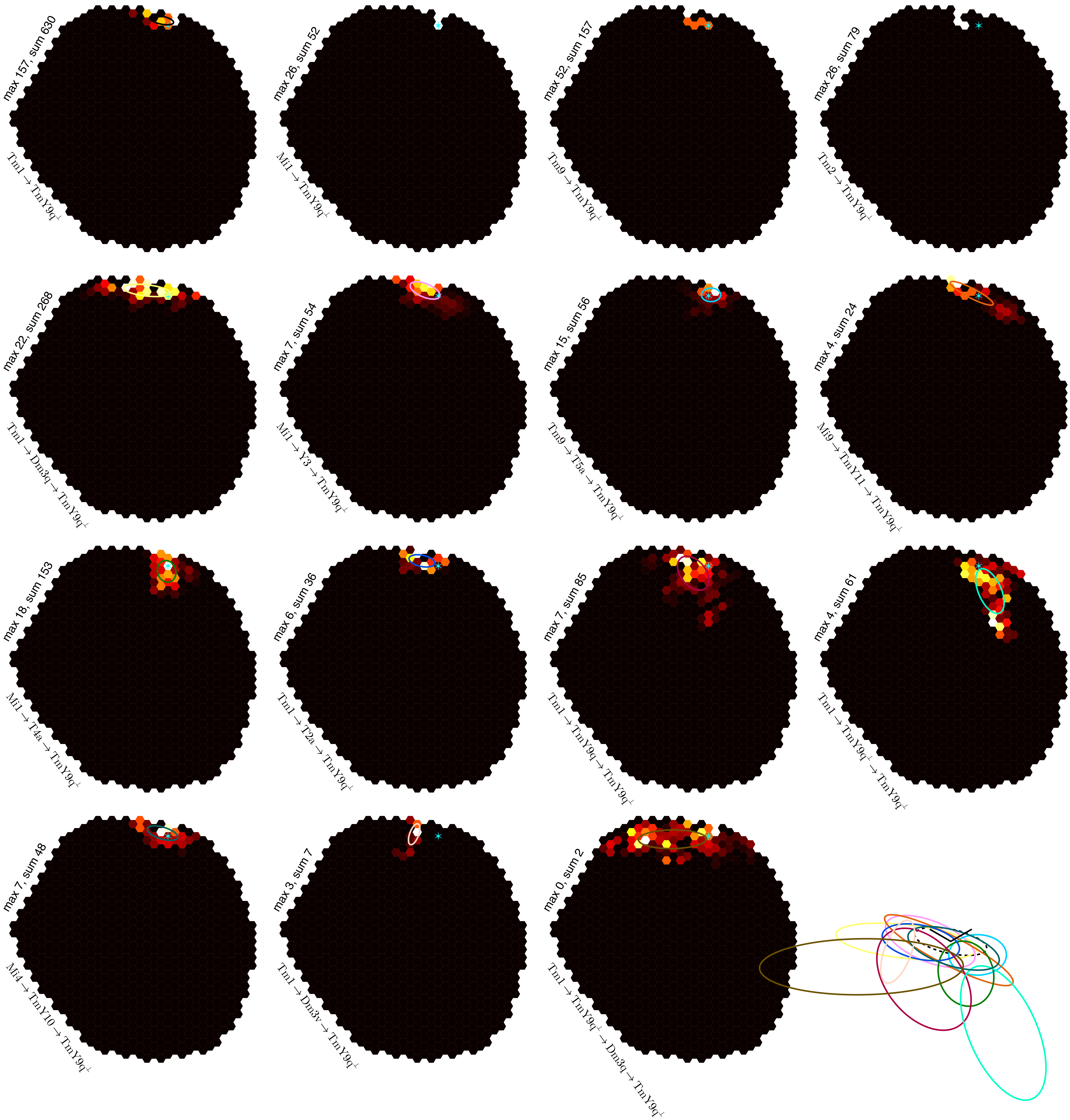
max 157, sum 630
Tm1 → TmY9q⊥
max 26, sum 52
Mi1 → TmY9q⊥
max 52, sum 157
Tm9 → TmY9q⊥
max 26, sum 79
Tm2 → TmY9q⊥
max 22, sum 268
Tm1 → Dm3q → TmY9q⊥
max 7, sum 54
Mi1 → Y3 → TmY9q⊥
max 15, sum 56
Tm9 → T5a → TmY9q⊥
max 4, sum 24
Mi9 → TmY11 → TmY9q⊥
max 18, sum 153
Mi1 → T4a → TmY9q⊥
max 6, sum 36
Tm1 → T2a → TmY9q⊥
max 7, sum 85
Tm1 → TmY9q → TmY9q⊥
max 4, sum 61
Tm1 → TmY9q⊥ → TmY9q⊥
max 7, sum 48
Mi4 → TmY10 → TmY9q⊥
max 3, sum 7
Tm1 → Dm3v → TmY9q⊥
max 0, sum 2
Tm1 → TmY9q⊥ → Dm3q → TmY9q⊥
